# Adherence to immunosuppressive therapy following liver transplantation: an integrative review

**DOI:** 10.1590/1518-8345.1072.2778

**Published:** 2016-08-29

**Authors:** Ramon Antônio Oliveira, Ruth Natália Teresa Turrini, Vanessa de Brito Poveda

**Affiliations:** 1 Master's Student, Escola de Enfermagem, Universidade de São Paulo, São Paulo, SP, Brazil.; 2 PhD, Associate Professor, Escola de Enfermagem, Universidade de São Paulo, São Paulo, SP, Brazil.; 3 PhD, Professor, Escola de Enfermagem, Universidade de São Paulo, São Paulo, SP, Brazil.

**Keywords:** Liver Transplantation, Medication Adherence, Patient Compliance, Nursing

## Abstract

**Objective:**

to investigate the evidence available in the literature on non-adherence to immunosuppressive therapy among patients undergoing liver transplantation.

**Method:**

integrative literature review, including research whose sample consisted of patients aged over 18 years undergoing liver transplantation. It excluded those containing patients undergoing multiple organ transplants. For the selection of articles, Medline / Pubmed, CINAHL, LILACS, Scopus and Embase were searched. The search period corresponded to the initial date of indexation of different bases, up to the deadline of February 10, 2015, using controlled and uncontrolled descriptors: liver transplantation, hepatic transplantation, liver orthotopic transplantation, medication adherence, medication non-adherence, medication compliance and patient compliance.

**Results:**

were located 191 investigations, 10 of which met the objectives of the study and were grouped into four categories, namely: educational process and non-adherence; non-adherence related to the number of daily doses of immunosuppressive medications; detection methods for non-adherence and side effects of therapy.

**Conclusion:**

there were risk factors related to the health service, such as control and reduction of the number of doses; related to the individual, such as being male, divorced, alcohol or other substances user, exposed to low social support and being mentally ill.

## Introduction

Liver transplantation allows the patients with terminal liver disease the opportunity to increase their quality of life, coupled with increased survival. However, the results are closely linked to the daily commitment of the patient with their immunosuppressive therapy^1^ ^-^ ^3^.

The graft receiver survival time may vary according to the initial diagnosis, ranging between 60 and 70% in the first five years, depending on the type of primary disease leading to the need of transplantation. It is worth noting that the procedure allows that approximately 80% of patients resume their work activities^4^.

It is clear that non-adherence to immunosuppressive therapy increases the risk of graft loss, in addition there is increased morbidity, represented by the presence of tremor, neurotoxicity, and acute renal failure and also the increase in mortality and re-hospitalization, raising the costs to the health system^3^ ^-^ ^5^.

Thus, adherence to the treatment plan can be defined as the patients' behavior, which meets the recommendations agreed with health professionals in relation to taking medicines, following the diet or changing their lifestyle^(6-7 )^.

Evaluation of adherence to drug therapy is a complex task. However, some methods have been proposed for their verification, ranging from questionnaires, measurement of serum drug dosage, or the counting of dispensed tablets; yet, it is noteworthy that none of these forms of assessment obtained greater sensitivity than 80%^8^.

The non-adherence rates to immunosuppressive medications among patients receiving solid organs can range from 36% among kidney transplant; 14.5% of heart recipients and 6.7% of liver transplant recipients. A previous research has shown that one of every 10 deaths from liver transplant patients is related to non-adherence to immunosuppressant^8^. On the other hand, a meta-analysis including 147 studies published between 1981 and 2005 addressed the adherence to immunosuppressive therapy following solid organ transplantation, and found that only 20% of these articles were related to liver recipients^9^.

Nurse is the professional spending more time with the patient, so he is the most suitable for the development of educational activities. The result of these actions reflected in increased satisfaction and quality of life, the effective delivery of care at home, anxiety reduction, the empowerment of the individual against the disease process and an increased treatment adherence^10^.

Thus, it is of great value to research the reasons that may contribute to non-adherence to treatment in the postoperative period, in order to support nursing actions with this group of patients.

Therefore, this study aimed to investigate evidence available in the literature on non-adherence to immunosuppressive therapy among patients undergoing liver transplantation.

## Method

For the preparation of the integrative review (IR) the following steps were followed: identifying the topic and defining the guiding question; sampling or literature search; extraction of data from the included studies; evaluation of studies and interpretation of results, and finally, the knowledge synthesis or integrative review of the presentation itself^11^.

**Figure 1:**
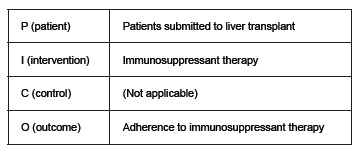
PICO strategy adopted to build the guiding question for the integrative review. Sao Paulo, 2015

For preparation the guiding question of the review, we used the PICO strategy^12^, described in sequence (Figure 1).

So the central question of this review consisted of: what factors indicated by the scientific literature, interfere with adherence in patients submitted to liver transplantation, to the therapy with immunosuppressant drugs?

The following inclusion criteria were: primary studies that included in the sample patients aged over 18 years undergoing liver transplantation; published in Portuguese, English or Spanish. Exclusion criteria were: scientific articles that addressed the issue of adherence among patients undergoing other types of transplants or multiple organ transplants.

Regarding the guiding question, the controlled and non-controlled descriptors were: *liver transplantation, hepatic transplantation, orthotopic liver transplantation, medication adherence, medication non-adherence, medication compliance and patient compliance*.

For the selection of articles included in the review the following databases were consulted: *Medical Literature Analysis and Retrieval System Online* (Medline / Pubmed), *Cumulative Index to Nursing and Allied Health Literature* (CINAHL-Ebsco) and *Latin American and Caribbean Health Sciences* (LILACS), Scopus and Embase. In the selection of descriptors the terms used are those in the *Medical Subject Headings* (MeSH), in the *List of Headings* of *CINAHL Information Systems* and Descriptors in Health Sciences (DeCS) of the Virtual Health Library. The pre-determined search period corresponded to the initial date of indexation of the different databases, up to the deadline of February 10, 2015.

**Figure 2:**
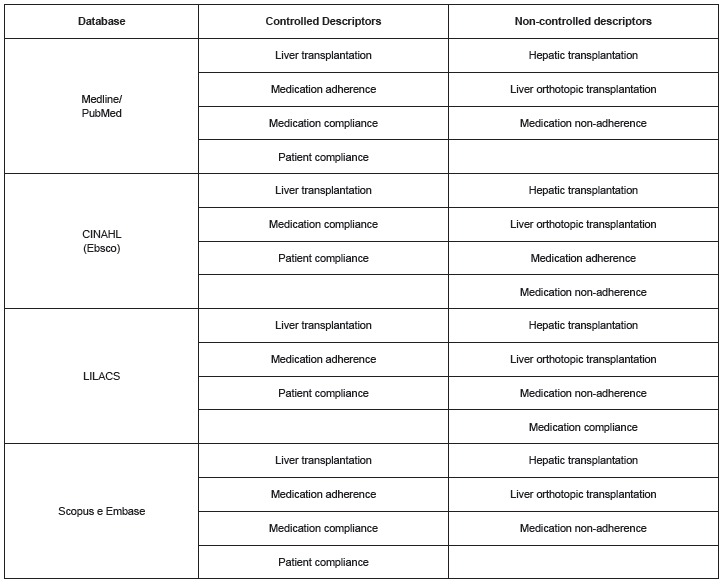
Controlled and non-controlled descriptors used for the review by each searched database. Sao Paulo, 2015

Thus, considering the analyzed databases, the descriptors used are described in the table below (Figure 2).

It is worth noting that several combinations among the descriptors cited previously were performed in order to ensure the maximum scope of search.

**Figure 3:**
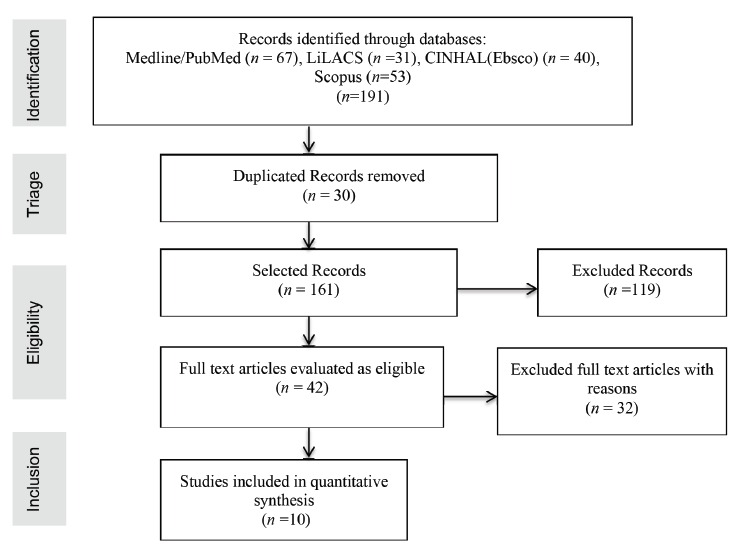
Diagram of inclusion and exclusion of articles available in the researched databases. Sao Paulo, 2015

The next figure describes the search process (Figure 3).

The search returned 191 articles in the databases selected for this study. Initially, we proceeded to read the titles and abstracts, checking the adequacy of the analyzed studies compared with the proposed inclusion criteria in this study. Of these, 30 records were excluded for being duplicates, 119 per inadequacy to the subject of the review, 20 included subjects under 18 years of age and 12 included patients undergoing multiple organ transplants.

**Figure 4:**
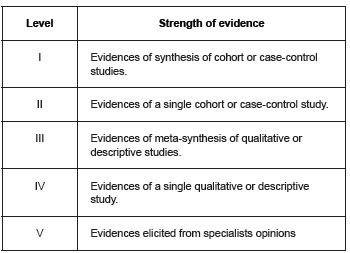
Classification of the strength of evidence for clinical questions for prognosis or prediction

Source: Melnyk, B.M. & Fineout-Overholt, E. (2014). Evidence-based practice in nursing and healthcare: A guide to best practice.3ª ed. USA: Wolters Kluwer (14)

The extraction of data items included was performed with the aid of a validated instrument^13^. For the analysis of the research study design and level of evidence it was used concepts proposed by Melnyk, Fineout-Overholt for clinical questions related to prognosis or prediction (Figure 4)^14^.

## Results

Replies to the proposed inclusion criteria 10 articles, published between 2005 and 2013, highlighting the year 2013 with four (40%) of these publications. Among the journals, Clinical Transplantation (30%) with three published investigations, Transplantation Proceedings with two (20%) and Transplant International, with two (20%) were the main sources.

All included investigations were published in the English language, nine of them (90%), written by medical professionals. As for the origin, five of them (50%) were produced in North America and five (50%) in European countries.

As for the strength of the evidence of the studies revealed that nine (90%) articles were classified as level two evidence, that is, cohort studies and one of the researches (10%) was considered at the level four evidence, a cross-sectional study.

**Figure 5:**
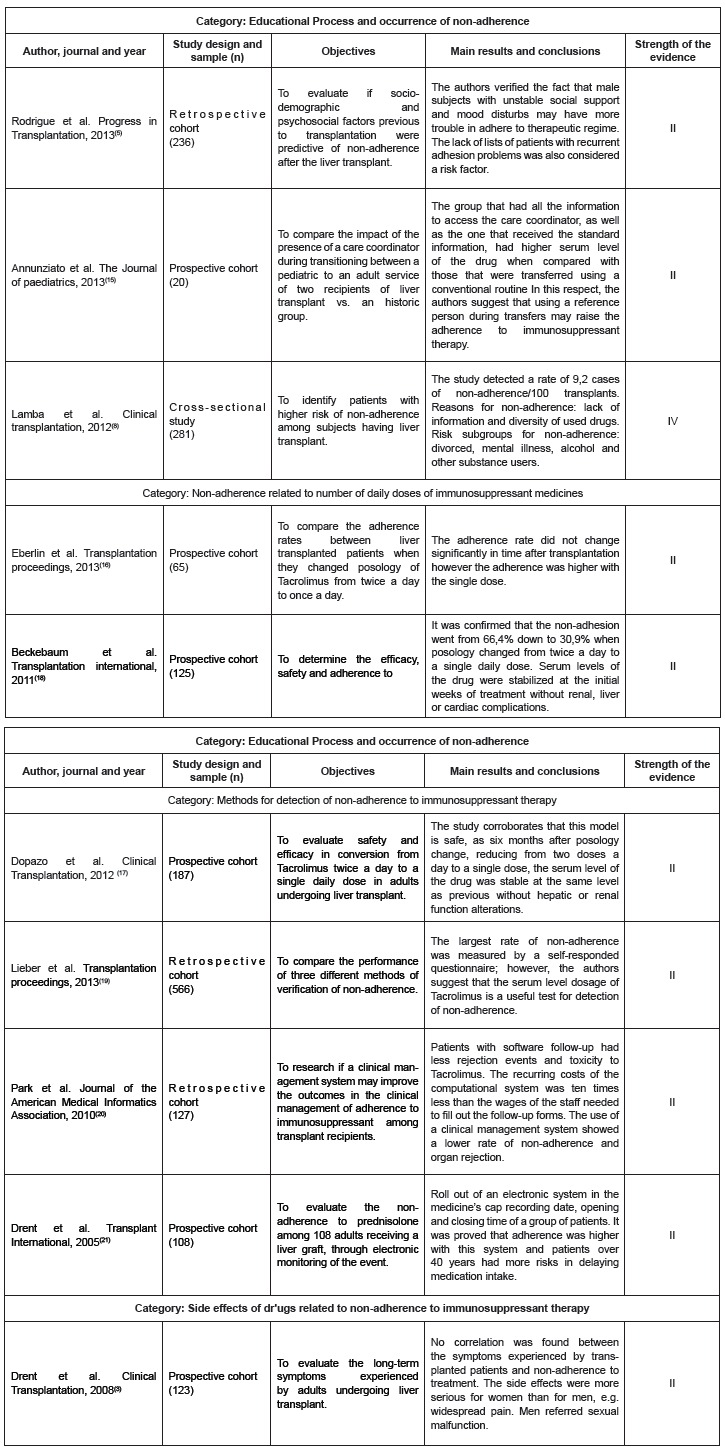
Display of articles following analysis categories, year of publication, journal, objectives, main results and conclusions. Sao Paulo, Brazil, 2015.

In order to facilitate the understanding of the highlighted results, the investigations included in this review were grouped together into categories, namely: educational process and the occurrence of non-compliance; non-adherence related to the number of daily doses of immunosuppressive medications; detection methods of non-adherence to immunosuppressive therapy and medication side effects related to non-adherence to immunosuppressive therapy (Figure 5).

## Discussion

The selected studies on the issue of non-adherence to immunosuppressive therapy in patients undergoing liver transplantation were produced predominantly in North America and Europe.

Another fact of equal relevance refers to the absence of studies by nursing professionals, since 90% of the articles included are the results of research carried out by professionals from other fields of knowledge. Regarding the methodological design, the majority of the sample consisted of cohort studies, a design that leads to difficulties in controlling bias^22^.

It is recognized that the best evidence is obtained by means of high quality methodological studies, allowing the generalization of the research results and also gathering information capable of providing support for clinical decision making, such as randomized controlled trials. However, it is noteworthy that the cohort studies are typically used to evaluate results of risk exposure^23^. Thus, this may be one of the designs recommended for research and evaluate risk factors for non-adherence to immunosuppressive therapy.

Therefore, the data found in different studies included in the present IR were synthesized into categories, the first one refers to the "Educational process and the occurrence of non-adherence" where the risk factors for non-adherence were of two kinds: related to the therapeutic process, such as lack of information on the treatment and the use of various drugs; and related to the individual, such as being male, alcohol users or other substances, exposed to lower or unstable social support and those with mental illnesses^5^ ^,^ ^8^ ^,^ ^15^. The strategies adopted among the studies included in this category suggest the optimization of the teaching-learning process, through the inclusion of family members training them in the use of drugs in the preoperative period^5^. In addition, direct contact with a professional responsible for the clinical follow-up seems to have a consequence in better adherence rates^15^.

Also noteworthy are the risk factors related to the organization of health services, such as the lack of records of patients with persistent compliance problems, since the formal ignorance of these subjects may result in inadequate planning of educational activities^5^.

Another category was "non-adherence related to the number of daily doses of immunosuppressive drugs," which addressed the adherence difficulties caused by the use of several drugs simultaneously. The intervention studied in this group of papers was the change in posology, from twice a day, for a daily single dose. The three studies included, found significant reduction of non-adherence, stabilization of serum drug levels and absence of liver, renal and cardiac complications^16^ ^-^ ^18^.

In the category "Methods of detection of non-adherence to immunosuppressive therapy," it was noted that putting in place surveillance methods for adherence, interferes in patients' response to drug therapy and therefore result in better outcomes of transplantation. However, there is no consensus regarding the best method for measurement of non-compliance; and three studies^19^ ^-^ ^21^ suggest that the self-administered questionnaire resulted in the measurement of the higher rate of non-compliance; another suggested tool is the implementation of a computer program for monitoring the use of medicines^20^, when associated with the assistance of health professionals, resulted in fewer rejection and intoxication events by the immunosuppressant drug. Another suggested method is an electronic device installed on the caps of the vials of drugs that would be able to detect the time of its opening and its closing^19^ ^-^ ^21^.

The last category was composed of a single work and refers to the evaluation of side effects related to immunosuppressive therapy as a risk factor for non-adherence^3^. This study found that the most serious side effects were widespread pain among female subjects, and among men sexual dysfunction, however, there was no correlation between symptoms perceived by patients and the phenomenon of non-adherence^3^.

Patients undergoing liver transplantation are patients with chronic diseases and require the constant use of drugs, with risks and health problems, making it imperative for them to be able to understand the importance of the procedure; learn to deal with the drugs; change hygiene habits, with the aim of combating infectious processes and adapt to various changes, such as the self-image, and social issues, such as work, for instance^11^.

In this sense, and due to the complexity in the treatment guidance, it was developed the "Guideline for evaluation of the indication of liver transplant" of the Association for the Study of Liver Disease and the American Transplant Society, which indicates the need, during the evaluation period of the indication of the transplant, to conduct meetings for patients and family education about all aspects involved in the procedure and even on adherence to immunosuppressive medications^1^.

In the research that evaluated 370 subjects undergoing transplants in France, 135 were liver transplant recipients and among them, it was found that 51% of subjects had non-adherence to treatment. It was stated also that the simplification of drugs protocols can increase compliance, because patients have difficulties in dealing with the treatment^24^.

In research^25^, which measured knowledge before and after the educational intervention among candidates to liver graft, it was found that despite the intervention contemplated the perioperative period, the understanding of the information related to the pre-operative period was higher compared to the post-operative^25^.

Another study sought to evaluate the effectiveness of the guidelines conducted by a multidisciplinary group for patients in the pre-operative period of liver transplantation, including 53 patients and 60 companions, showing that after the meetings of the educational group, there was an increase in the number of right answers in almost all areas covered, and the item with the highest increase in the number of correct answers was related to the use of immunosuppressive drugs^26^.

Several factors have been associated with behaviors of non-compliance, ranging from health problems such as depression^27^, to questions related to social status or associated with intrinsic characteristics of patients, such as being male, with low social support^5^, or related to the health system, such as the lack or absence of available medication^9^. This last factor in particular, does not apply in theory to Brazil, since health care is provided for in the Constitution of 1988, which states that health "is everyone's right and duty of the state", therefore immunosuppressive drugs are offered by the Brazilian National Health System (SUS)^28^.

Another aspect that may contribute to non-adherence are the side effects, which seem to cause more discomfort among women than among men, ranging from widespread pain, more commonly reported by women, to sexual dysfunction (in men)^3^. Studies including patients with the human immunodeficiency virus (HIV), found that the absence of side effects is a protective factor for non-adherence to medication^29^ ^-^ ^30^.

The reduction in the dosage of immunosuppressant to a single daily dose is considered as a measure that confirms the reduction of the problem of non-compliance^17^ ^-^ ^19^, an aspect pointed out by studies, showed reduction of non-adherence to immunosuppressive therapy when there was an increase in dose and a reduction in dosage for a single daily dose. Other research developed involving subjects with chronic non-communicable diseases have shown that the reduction in the number of drug doses, and the dosage corresponding to the routine of patients resulting in increased adherence to therapy^31^ ^-^ ^32^.

Many methods can contribute to the detection of non-adherence to immunosuppressive therapy, from self-completed questionnaires, measurement of serum drug level and use of electronic systems that are able to detect the opening and closing of medicine bottles, generating reports to health professionals who monitor the patients^16^ ^,^ ^20^.

There is no consensus regarding the best method of assessment of non-compliance. Probably this actually happens since it is a multidimensional phenomenon, and for this reason should be evaluated in various ways, including the use of technology^30^ ^,^ ^33^ ^-^ ^35^.

Therefore, it is remarked the importance of guidance, through health education in relation to adherence to immunosuppressive therapy. Thus, we highlight the role of the nurse in the development of these activities, promoting safe behavior and the use of mechanisms that favor adherence in relation to immunosuppressive drugs.

It is believed that the issues brought to light in this study are able to guide the decision making process of nurses and health professionals in order to map out and know which patients are more prone to non-adherence, suggesting strategies to better monitoring and increasing adherence to therapy, but ultimately preventing and reducing the episodes of rejection, re-hospitalization and especially damage to the health and quality of life of the patients^25^.

## Conclusion

It was concluded that the risk factors for non-adherence to immunosuppressive medications among adult patients undergoing liver transplantation, pointed out by the analyzed scientific literature are of two kinds: those related to health services, characterized by the absence of lists for customized monitoring of patients which are not adherent, no clear methodology enabling the classification of patients into adherent and non-adherent groups and personal risk factors evidenced by problems or lack of social support, lack of information, simultaneous use of several drugs, diagnosis of mental illness, alcohol and other drugs use and being male.

The literature also points out, as protective factors for the phenomenon of non-adherence, the follow-up by the family in therapeutic encounters that provide health education; receiving standardized information; direct contact with the adequate type of professional, responsible for the clinical monitoring of the patient and a reduction of the dose to once daily.

Limitations of this study include the lack of investigations with greater methodological rigor and allowing larger comparisons and extrapolation of the results.

There was also remarkable the absence of national articles on the theme, indicating a field to be explored by the Brazilian scientific literature, as it becomes interesting to know what are the factors that impact on adherence to immunosuppressive therapy among Brazilian patients since the health care organization in Brazil differs from countries whose papers were included in this review.

## References

[1] Martin P, DiMartini A, Feng S, Brown RS, Fallon M. (2014). Evaluation for Liver Transplantation in Adults: 2013 Practice Guideline by the American Association for the Study of Liver Diseases and the American Society of Transplantation. Hepatology. (Baltim).

[2] Ming-Ming X, Jr. Brown RS (2015). Liver Transplantation for the Referring Physician. Clin Liver Dis.

[3] Drent G, Moons P, De Geest S, Kleibeuker JH, Haagsma EB (2008). Symptom experience associated with immunosuppressive drugs after liver transplantation in adults Possible relationship with medication non-compliance?. Clin Transplant.

[4] Xu L, Xu MQ, Yan LN, Li B, Wen TF, Wang WT (2012). Causes of mortality after liver transplantation a single center experience in mainland China. Hepatogastroenterology.

[5] Rodrigue JR, Nelson DR, Hanto DW, Reed AI, Curry MP (2013). Patient-reported immunosuppression nonadherence 6 to 24 months after liver transplant association with pretransplant psychosocial factors and perceptions of health status change. Progr Transplant.

[6] World Health Organization (WHO) (2003). Adherence to long-term therapies: evidence for action.

[7] Bender BG. (2014). Can Health Care Organizations Improve Health Behavior and Treatment Adherence?. Popul Health Manage..

[8] Lamba S, Nagurka R, Desai KK, Chun SJ, Holland B, Koneru B (2012). Self-reported non-adherence to immune-suppressant therapy in liver transplant recipients demographic, interpersonal, and intrapersonal factors. Clin Transplant.

[9] Dew MA, DiMartini AF, De Vito Dabbs A, Myaskovsky L, Steel J, Unruh M (2007). Rates and risk factors for nonadherence to the medical regimen after adult solid organ transplantation. Transplantation.

[10] Jaarsma T, Nikolova-Simons M, Wal MHL. (2012). Nurses' strategies to address self-care aspects related to medication adherence and symptom recognition in heart failure patients: An in-depth look. Heart Lung.

[11] Mendes KDS, Silveira RCCP, Galvão CM (2008). Integrative Literature Review a research method to incorporate evidence in health care and nursing. Texto Contexto Enferm.

[12] Santos CMC, Pimenta CAM, Nobre MRC. (2007). The PICO strategy for the research question construction and evidence search. Rev. Latino-Am. Enfermagem.

[13] Ursi ES, Galvão CM (2006). Perioperative prevention of skin injury an integrative literature review. Rev. Latino-Am. Enfermagem.

[14] Melnyk BM, Finout-Overholt E (2014). Evidence-based practice in nursing & healthcare.

[15] Annunziato RA, Baisley MA, Arrato N, Barton C, Henderling F, Arnon R (2013). Strangers Headed to a Strange Land A Pilot Study of Using a Transition Coordinator to Improve Transfer from Pediatric to Adult Services. J Pediatr.

[16] Eberlin M, Otto G, Krämer I (2013). Increased medication compliance of liver transplant patients switched from a twice-daily to a once-daily tacrolimus-based immunosuppressive regimen. Transplant Proc.

[17] Dopazo C, Rodriguez R, Llado L, Calatayud D, Castells L, Ramos E (2012). Successful conversion from twice-daily to once-daily tacrolimus in liver transplantation observational multicenter study. Clin Transplant.

[18] Beckebaum S, Lacob S, Sweid D, Sotiropoulos GC, Saner F, Kaiser G (2011). Efficacy, safety, and immunosuppressant adherence in stable liver transplant patients converted from a twice-daily tacrolimus-based regimen to once-daily tacrolimus extended-release formulation. Transplant Int.

[19] Lieber SR, Volk ML (2013). Non-adherence and graft failure in adult liver transplant recipients. Dig Dis Sci.

[20] Park ES, Peccoud MR, Wicks KA, Halldorson JB, Carithers RL, Reyes JD (2010). Use of an automated clinical management system improves outpatient immunosuppressive care following liver transplantation. J Am Med Inform Assoc.

[21] Drent G, Haagsma EB, De Geest S, Van Den Berg AP, Ten Vergert EM, van den Bosch HJ (2005). Prevalence of prednisolone (non)compliance in adult liver transplant recipients. Transpl Int.

[22] Carlson MDA, Morrison RS. (2009). Study Design, Precision, and Validity in Observational Studies. J Palliat Med.

[23] Lazcano-Ponce E, Fernández E, Salazar-Martínez E, Hernández-Ávila M. (2000). Estudios de cohorte. Metodología, sesgos y aplicación. Salud Pública Méx..

[24] Dharancy S, Giral M, Tetaz R, Fatras M, Dubel L, Pageaux G-P. (2012). Adherence with immunosuppressive treatment after transplantation: results from the French trial PREDICT. Clin Transplant.

[25] Mendes KDS, Silva OC, Ziviani LC, Rossin FM, Zago MMF, Galvão CM. (2013). Educational intervention for liver transplantation candidates. Rev. Latino-Am. Enfermagem..

[26] Guimaro MS, Lacerda SS, Bacoccina TD, Karam CH, de Sá JR, Ferraz-Neto BH (2007). Evaluation of Efficacy in a Liver Pretransplantation Orientation Group. Transplant Proc.

[27] Bautista LE, Vera-Cala LM, Colombo C, Smith P. (2012). Symptoms of depression and anxiety and adherence to antihypertensive medication. Am J Hypertens..

[28] Constituição, 1988 (BR) (1988). Constituição da República Federativa do Brasil.

[29] Nachega JB, Morroni C, Zuniga JM, Schechter M, Rockstroh J, Solomon S (2012). HIV Treatment Adherence, Patient Health Literacy, and Health Care Provider-Patient Communication: results from the 2010 AIDS Treatment for Life International Survey. J Int Assoc Physicians AIDS Care.

[30] Al-Dakkaka I, Patela S, McCanna E, Gadkarib A, Prajapatib G, Maieseb EM. (2013). The impact of specific HIV treatment-related adverse events on adherence to antiretroviral therapy: A systematic review and meta-analysis. AIDS Care.

[31] Toha MR, Teoa V, Kwana YH, Raaja S, Tanc SD, Tan JZY. (2014). Association between number of doses per day, number of medications and patient's non-compliance, and frequency of readmissions in a multi-ethnic Asian population. Prev Med Report.

[32] Tsai KT, Chen JH, Wen CJ, Kuo HK, Lu IS, Chiu LS (2012). Medication Adherence Among Geriatric Outpatients Prescribed Multiple Medications. Am J Geriatr Pharmacother.

[33] Lehmann A, Aslani P, Ahmed R, Celio J, Gauchet A, Bedouch P (2014). Assessing medication adherence: options to consider. Int J Clin Pharm.

[34] Williams AB, Amico KR, Bova C, Womack JA. (2013). A Proposal for Quality Standards for Measuring Medication Adherence in Research. AIDS Behav.

[35] Montesa JM, Medinab E, Gomez-Beneytoc M, Maurinob J. (2012). A short message service (SMS)-based strategy for enhancing adherence to antipsychotic medication in schizophrenia. Psychiatry Res..

